# Kinin B1R Activation Induces Endoplasmic Reticulum Stress in Primary Hypothalamic Neurons

**DOI:** 10.3389/fphar.2022.841068

**Published:** 2022-03-08

**Authors:** Acacia White, Rohan Umesh Parekh, Drew Theobald, Pranaya Pakala, Ariel Lynn Myers, Rukiyah Van Dross, Srinivas Sriramula

**Affiliations:** Department of Pharmacology and Toxicology, Brody School of Medicine at East Carolina University, Greenville, NC, United States

**Keywords:** kinin B1 receptor, unfolded protein response, neurons, hypertension, endoplasmic reticulum stress (ER stress)

## Abstract

The endoplasmic reticulum (ER) is a key organelle involved in homeostatic functions including protein synthesis and transport, and the storage of free calcium. ER stress potentiates neuroinflammation and neurodegeneration and is a key contributor to the pathogenesis of neurogenic hypertension. Recently, we showed that kinin B1 receptor (B1R) activation plays a vital role in modulating neuroinflammation and hypertension. However, whether B1R activation results in the progression and enhancement of ER stress has not yet been studied. In this brief research report, we tested the hypothesis that B1R activation in neurons contributes to unfolded protein response (UPR) and the development of ER stress. To test this hypothesis, we treated primary hypothalamic neuronal cultures with B1R specific agonist Lys-Des-Arg^9^-Bradykinin (LDABK) and measured the components of UPR and ER stress. Our data show that B1R stimulation *via* LDABK, induced the upregulation of GRP78, a molecular chaperone of ER stress. B1R stimulation was associated with an increased expression and activation of transmembrane ER stress sensors, ATF6, IRE1α, and PERK, the critical components of UPR. In the presence of overwhelming ER stress, activated ER stress sensors can lead to oxidative stress, autophagy, or apoptosis. To determine whether B1R activation induces apoptosis we measured intracellular Ca^2+^ and extracellular ATP levels, caspases 3/7 activity, and cell viability. Our data show that LDABK treatment does increase Ca^2+^ and ATP levels but does not alter caspase activity or cell viability. These findings suggest that B1R activation initiates the UPR and is a key factor in the ER stress pathway.

## Introduction

Cardiovascular disease continues to be the leading cause of death worldwide, and despite the use of preventative measures such as lifestyle changes and aggressive hypertension treatment options that are currently available, the incidence and mortality rate remains high. Hypertension is considered a primary risk factor for the development of cardiovascular diseases and has recently been associated with the development of neurodegenerative conditions such as Alzheimer’s, Parkinson’s and Huntington’s disease (Bergantin, 2019; Steventon et al., 2020; Youwakim and Girouard, 2021). Surmounting evidence indicates chronic inflammation is a vital and common pathophysiological trait for the development of both cardiovascular and neurodegenerative diseases. Chronic inflammation, at the peripheral and central nervous system levels, contributes to the physiopathology of hypertension via an increased sympathetic drive, neuroinflammation and elevated oxidative stress within cardiovascular regulatory regions of the brain, such as the hypothalamic paraventricular nucleus (PVN) (Mann, 2018). Inflammation is often associated with the etiology of many diseases such as cancer, because inflammation can potentiate misfolding of mutated proteins and cause the accumulation of unfolded proteins, a process known to induce endoplasmic reticulum (ER) stress. The ER is one of the largest organelles in a cell and is known for coordinating protein synthesis, folding and transport (Schwarz and Blower, 2016). When this process is disrupted, the accumulation of misfolded proteins results in ER stress, and if unresolved, can result in apoptotic cell death (Lam et al., 2020).

The ER contains high concentrations of foldases, protein chaperones, ATP, and Ca^2+^, which are primarily used for protein folding, post-translational modifications, and sensing homeostatic deviations in the ER (Ma and Hendershot, 2004; Phillips and Voeltz, 2016; Lin et al., 2021). Physiological stressors such as mutated and misfolded proteins, a depletion of intracellular Ca^2+^ and ATP supply, and reactive oxygen species (ROS) accumulation can impair ER function and initiate the unfolded protein response (UPR) (Ma and Hendershot, 2004; Lin et al., 2008). The UPR is a cellular response mechanism which is initiated to alleviate stress and disequilibrium within the cell to reduce unfolded protein load to maintain cell viability and function. The UPR is a signal transduction pathway that is initiated by the dissociation of ER-localized protein sensors: inositol-requiring 1*α* (IRE1*α*), PKR-like endoplasmic reticulum kinase (PERK) and activating transcription factor 6 (ATF6) from the protein chaperone, glucose regulated protein 78 (GRP78 or BiP) (Kadowaki and Nishitoh, 2013; Tsai et al., 2015). ATF6 activation results in translocation from the ER to the Golgi apparatus where it is cleaved. Once cleaved, ATF6 is then translocated into the nucleus, increasing the transcription of inflammatory genes (Kadowaki and Nishitoh, 2013). IRE1α activation results in the splicing of X-box binding protein 1 (XBP1) mRNA and the synthesis of XBP1s (XBP1 spliced), a transcription factor which upregulates UPR and inflammatory response genes (Lubamba et al., 2015; Adams et al., 2019). When PERK is activated, it phosphorylates translation-initiation factor2*α* (eIF2*α*), which promotes cellular survival or death (Rozpedek et al., 2016). Alternatively, if the ER is unable to reestablish ER homeostasis, then apoptotic pathways, mediated by C/EBP homologous proteins (CHOP) and caspases, become activated (Oyadomari and Mori, 2004).

Recent studies have shown that cellular disturbances known to contribute to ER stress, including dysfunctional alterations in intracellular Ca^2+^ concentrations, accumulation of misfolded or mutated proteins, neuronal apoptosis and oxidative stress, are prevalent in neurogenic hypertension and neurodegenerative diseases (Lindholm et al., 2006; Hosoi and Ozawa, 2009; Young, 2017). Due to the widespread adverse cellular effects of ER stress, organelles such as the mitochondria experience increased ROS production, therefore proper ER functioning is vital to cellular survival and function. In neurons, ER stress causes neuroplastic changes, including alterations to synaptic transmission and neuronal signaling, leading to neurodegeneration, a primary mediator of neurogenic hypertension (Young, 2017). The brain is a key regulator of blood pressure and several studies have shown that ER stress in brain regions involved in autonomic blood pressure regulation, such as the subfornical organ, mediates hypertension (Young et al., 2015; Dai et al., 2016). However, whether ER stress in the brain, specifically in neurons, plays a role in these diseases is not yet known.

The kallikrein-kinin system (KKS) is a family of vasoactive peptides that play an important role in functional homeostasis and inflammation (Leeb-Lundberg et al., 2005). The physiological effects of kinins are mediated by two G-protein coupled receptor subtypes, kinin B1 (B1R) and B2 (B2R) receptors. The nature of these two receptors is distinct, with B2R being constitutively expressed and B1R expression being upregulated in the presence of inflammation and stress (Leeb-Lundberg et al., 2005). Unlike the B2R, once B1R is activated, it does not undergo desensitization or internalization, and its activation results in intracellular Ca^2+^ release (Austin et al., 1997; Lau et al., 2020). Previous studies from our laboratory and others have shown that neurohumoral regulatory regions of the brain such as the paraventricular nucleus of the hypothalamus play an important role in cardiovascular homeostasis (Sriramula et al., 2011; Sriramula and Lazartigues, 2017; Basting et al., 2018). Specifically, renin-angiotensin system overactivity, and inflammation in these regions of the brain contribute to sympathoexcitation and can result in the progression of cardiovascular diseases such as hypertension (Sriramula et al., 2011; Sriramula and Lazartigues, 2017). To further study the importance of these brain regions, we have standardized a primary hypothalamic neuronal culture model in mice. We showed that kinin B1R blockade attenuates Ang II induced neuroinflammation and oxidative stress in primary neurons (Parekh et al., 2020). More recently, we showed that activation of B1R upregulates ADAM17 and results in ACE2 shedding in primary hypothalamic neurons (Parekh and Sriramula, 2020). Interestingly, emerging evidence indicate that ER stress in the brain is a mediator of sympathoexcitation and hypertension (Young, 2017). We have previously demonstrated the increased expression of B1R in the hypothalamic paraventricular neurons and showed that blockade of B1R activation in the brain mitigates blood pressure elevation (Sriramula and Lazartigues, 2017). However, the role of B1R activation and its contribution to UPR and ER stress in neurons is not known. Thus, in this brief research report, using mouse primary hypothalamic neuronal cultures, we investigated whether B1R stimulation contributes to ER stress and causes UPR.

## Materials and Methods

### Hypothalamic Primary Neuronal Cell Cultures

Primary neurons were cultured from neonatal, or 1-day-old mice pups as previously described (Parekh et al., 2020; Parekh and Sriramula, 2020; Parekh et al., 2021). The experimental protocols used for breeding mice were approved by East Carolina University Animal Care and Use Committee (AUP #W261) and were performed in accordance with the National Institutes of Health Guidelines for the Care and Use of Laboratory Animals. Briefly, mouse pups were deeply anesthetized with isoflurane (4%) in an oxygen flow (1 L/min) before decapitation and brains were collected. Hypothalamic tissue was dissected, collected, and minced into small pieces using a sterile blade. Minced tissue was digested with HBSS containing 1% trypsin (T1426 Sigma-Aldrich, St. Louis, MO, United States) and 1.5 kU/mL DNaseI (D5025 Sigma-Aldrich) for 10 min at 37°C. Following the disassociation, the cells were spun down by centrifugation and resuspended in complete Neurobasal culture medium supplemented with 2% B27, 0.5 mM GlutaMax and penicillin/streptomycin (100 U/mL and 100 μg/ml, respectively) (Gibco). Dissociated neurons were then plated into poly-L-lysine-coated cell culture plates at a density of 50,000 cells per well (6-well plates), 25,000 cells per well (12-well plates), and 12,500 cells per well (96-well plates). The neurons were grown in a humidified atmosphere of 5% CO_2_–95% air at 37°C and treated with cytosine arabinofuranoside (Ara-C, 2 μM, C1768 Sigma-Aldrich) to arrest the growth of non-neuronal cells. Hypothalamic neuronal cultures were validated previously using immunofluorescence labeling with a neuron-specific cytoskeletal marker, MAP2 (microtubule associated protein 2) and a glial cell-specific marker, GFAP (glial fibrillary acidic protein) (Parekh et al., 2020). The neurons cultured with Ara-C treatment showed predominantly neuronal population, demonstrating numerous processes and discrete cellular morphology of neurons with cell-cell interaction (Parekh et al., 2020). Hypothalamic primary neurons were cultured for at least 10 days and then used for further experiments. The treatment durations and doses of Lys-[des-Arg^9^]-Bradykinin (LDABK, #3225, Tocris Bioscience, 300 nM) and Thapsigargin (#T9033, Sigma, 10 μM) are based on our preliminary studies and published literature (Oslowski and Urano, 2011; Parekh et al., 2020; Parekh and Sriramula, 2020).

### Immunofluorescence Staining

Primary neurons were grown on poly-L-lysine-coated glass cover slips in 12-well plates and fixed with 4% paraformaldehyde for 15 min. The cells were permeabilized with 0.1% Triton X-100 in 1 × PBS for 15 min and blocked with 5% donkey serum in 1 × PBS containing 0.1% Tween 20 for 1 h. Then, cells were incubated overnight at 4°C with primary antibodies against GRP78 (ab213258, abcam, 1:500) and ATF6 (IMG273, Imgex, 1:500). The next day, cells were washed three times with PBS +0.3% Tween-20 and incubated with appropriate Alexa Fluor conjugated secondary antibodies (Life Technologies, 1:1,000 dilution) for 1 h at room temperature, followed by DAPI or Lamin nuclear stain. Sections were mounted with ProLong Diamond Anti-Fade Mount (Invitrogen). Images were captured using an Echo Revolve Microscope.

### Protein Analysis by Western Blot

Western blots were performed on primary hypothalamic neuronal homogenates, as described previously (Parekh and Sriramula, 2020). Neuron samples were homogenized in 1× lysis buffer containing protease and phosphatase inhibitors cocktail (Roche) and centrifuged at 12,000×*g* and 4°C for 15 min. Protein concentration was determined using BCA protein assay kit (Thermo Fisher/Pierce). Equal amount (15 µg) of protein lysates were resolved on 4–15% or Any KD Mini-PROTEAN TGX gels (Bio-Rad) under reducing conditions and blotted on to PVDF membranes using *Trans*-Blot Turbo Transfer system (Bio-Rad). Membranes were blocked with Intercept-TBS blocking buffer (Licor) and immunoblotted overnight at 4°C with validated antibodies (1:1,000 dilution) against ATF6 (40256SS, Novus Biologicals), pIRE1*α* (ab48187, abcam), IRE1*α* (CS32945, Cell Signaling), pPERK (CS3179S, Cell Signaling), PERK (CS3192S, Cell Signaling), eIF*α*2 (CS9722S, Cell Signaling), peIF*α*2 (CS9721L, Cell Signaling), and GRP78 (ab213258, abcam). After washing, the membranes were incubated with IRDye secondary antibodies and imaged using Odyssey CLx imaging system (Licor). The blots were reprobed with GAPDH (1:1,000, #MAB374, Millipore Sigma) to confirm equal loading. The density of protein bands was quantitatively analyzed by ImageJ software (NIH) and expressed as a relative ratio against the loading control or corresponding total proteins.

### XBP Splicing Assay

RNA was isolated with Trizol Reagent and treated with DNase I followed by first strand synthesis with MMLV reverse transcriptase (Invitrogen). Reverse-transcriptase polymerase chain reaction (RT-PCR) was carried out using standard procedures. XBP1 forward 5′-GAA​CCA​GGA​GTT​AAG​AAC​ACG-3′ and reverse 5′-AGG​CAA​CAG​TGT​CAG​AGT​CC-3′ primers amplify both unspliced (205 bp) and spliced (179 bp) XBP1. GAPDH forward and reverse primers are 5′-GTC​TAC​TGG​TGT​CTT​CAC​CA-3′ and 5′-GTG​GCA​GTG​ATG​GCA​TGG​AC3′, respectively. PCR products were resolved on a 3% agarose gel using electrophoresis (Cambrex BioScience) and images were captured using gel doc system (Bio-Rad).

### MTS Cell Viability Assay

Primary neurons were plated in 96-well plates at a density of 12,500 cells per well and cultured for at least 10 days. The neurons are treated with LDABK (300 nM, 1, 5, 10 μM) and Thapsigargin (10 μM) for 24 and 48 h. MTS reagent (Promega, Madison, WI, United States) was then added to each well and the absorbance at 495 nm was measured according to the manufacturer’s instructions. Absorbance readings were acquired using the Infinite 200 Pro plate reader (Tecan Trading AG, Switzerland). Results were shown as percent of viable cells compared to control.

### Caspase 3/7 Activity Assay

Primary neurons were plated in 96-well plates at a density of 12,500 cells per well and cultured for at least 10 days. The neurons were treated with LDABK (300 nM, 1 μM, 5 μM, 10 μM) and Thapsigargin (10 μM) for 24 and 48 h. Caspase-Glo 3/7 reagent (Promega, Madison, WI, United States) was added to each well as directed by the manufacturer. The Caspase-Glo 3/7 kit measures the activity of the executioner caspases three and seven using the luminogenic substrate, Z-DEVD-aminoluciferin. Luminescence was measured using the Infinite 200 Pro plate reader (Tecan Trading AG, Switzerland). Results were shown as fold change in comparison to control.

### Extracellular ATP Measurement

ATP levels in the culture medium were quantified using the CellTiter-Glo 2.0 assay kit (Promega, Madison, WI, United States) according to the manufacturer’s instructions. Luciferin is monooxygenated by the luciferase enzyme in the presence of ATP producing luminescent signal. Therefore, the CellTiter-Glo 2.0 kit measures the generation of luminescent substrate. The intensity of the luminescent signal is proportional to the amount of ATP that is released into the culture medium by the cells. Luminescence was measured using the Infinite 200 Pro plate reader (Tecan Trading AG, Switzerland).

### Intracellular Ca^2+^ Measurement

Ca^2+^ levels were measured using the Fluo-4 NW Calcium Assay Kit from (Molecular Probes, ThermoFischer Scientific, Waltham, MA). Briefly, 100 μl Fluo-4-NW-dye-mix was added to each well and incubated for 30 min at 37°C, followed by 30 min incubation in the dark at room temperature. Changes in fluorescence from the Fluo-4-NW-dye quantify changes in intracellular calcium concentrations (excitation/emission 485/535 nm) using the Infinite 200 Pro plate reader (Tecan Trading AG, Switzerland).

### Statistics

Statistical analyses were performed using GraphPad Prism 7 (GraphPad Software). Data are presented as mean ± standard error of the mean (SEM). Multiple comparisons were made using 1-way analysis of variance followed by Tukey’s multiple comparisons test. Differences were considered statistically significant at *p* < 0.05.

## Results

### B1R Activation Increases GRP78 Expression in Primary Hypothalamic Neurons

**FIGURE 1:**
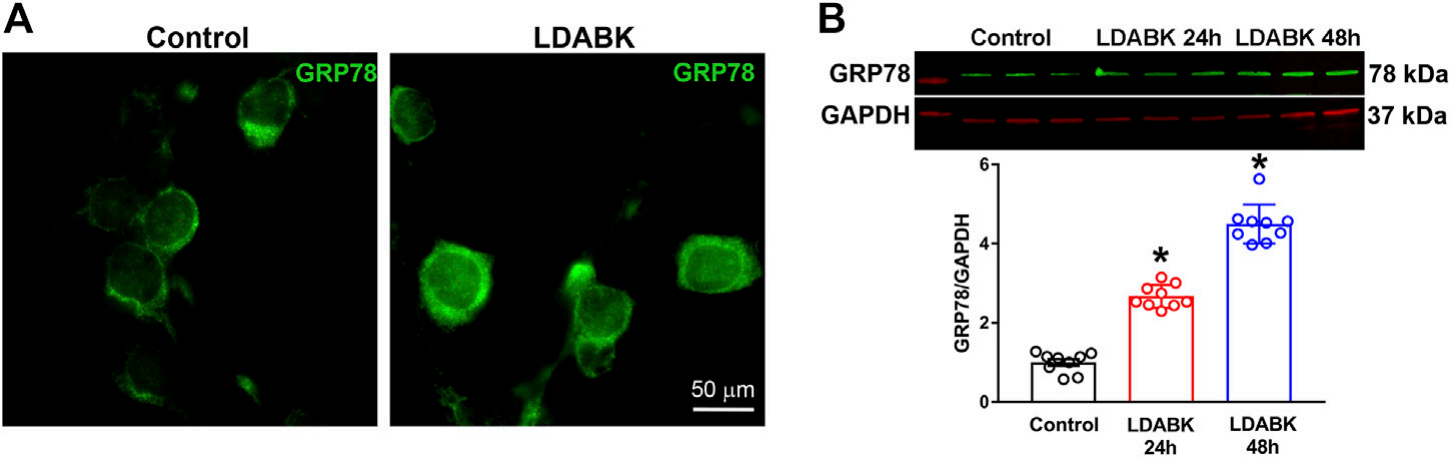
B1R activation increases GRP78 expression in primary hypothalamic neurons. **(A)** Immunofluorescence staining of GRP78 showing treatment with B1R specific agonist, LDABK (300 nM), increased protein expression of GRP78 in primary hypothalamic neurons. **(B)** Quantification of GRP78 protein expression using western blot analysis and quantification data shows a significant increase of GRP78 protein expression in LDABK (300 nM) treated neurons compared to vehicle (phosphate buffered saline) treated control neurons. *p < 0.05, compared to control. (*n* = 9/group).

Induction of ER-resident chaperone GRP78 is a fundamental indicator of stress in the ER lumen, and that the UPR signaling pathways are activated (Rao et al., 2002). To evaluate whether activation of B1R can induce ER stress, we first analyzed the expression of GRP78 in primary hypothalamic neuronal cultures treated with B1R specific agonist LDABK (300 nM) or its vehicle (PBS) as a control. The immunoreactivity of GRP78 was elevated in primary neurons treated with LDABK compared to control (Figure 1A). Analyzing protein expression by western blot analysis of the lysates from primary cultures, showed that GRP78 protein expression was increased by LDABK treatment at 24 and 48 h (Figure 1B). These results indicate that B1R stimulation results in GRP78 activation in neurons indicative of ER stress.

### B1R Activation Induces ER Stress Sensor Activation in Primary Hypothalamic Neurons

**FIGURE 2:**
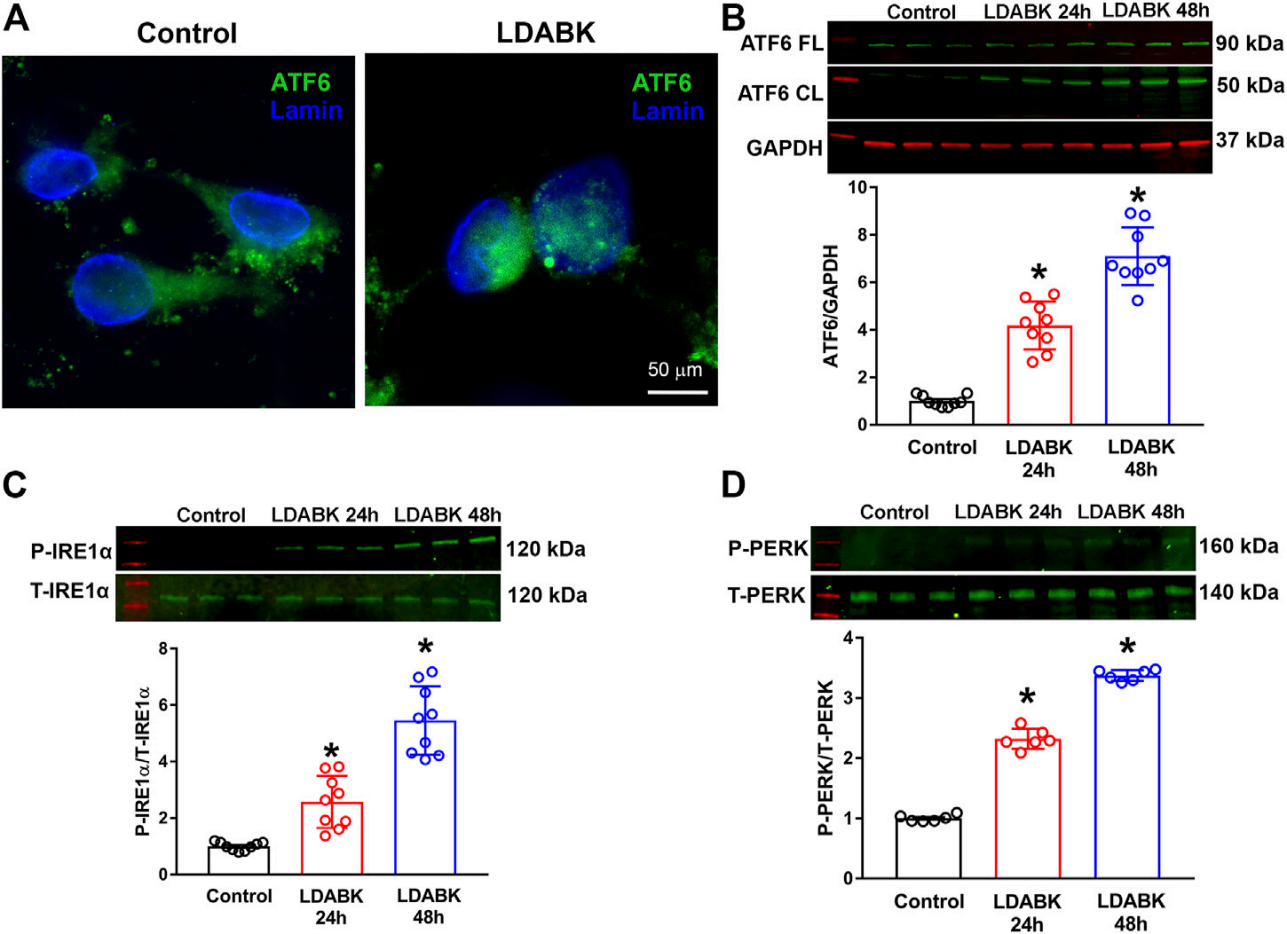
B1R stimulation induces the activation of ER stress sensors in primary hypothalamic neurons. Primary hypothalamic neurons were stimulated with B1R specific agonist LDABK (300 nM) or vehicle (phosphate buffered saline) for 24 or 48 h and the activation of ER stress sensors determined using immunofluorescence and western blot analysis. **(A)** Immunofluorescence staining revealed that ATF6 undergoes nuclear translocation in neurons after treatment with B1R agonist LDABK for 24 h. **(B)** Quantitative western blot analysis shows an increase in the activated cleaved form of ATF6 by LDABK stimulation. FL: Full length, CL: Cleaved. **(C)** Western blot analysis illustrates an increase in the phosphorylation of IRE1α and **(D)** PERK, following LDABK treatment in primary hypothalamic neurons. *p < 0.05, compared to control. (*n* = 6–9/group).

Activation of GRP78 coordinates the UPR by regulating the three main effector proteins: ATF6, IRE1*α*, and PERK (Wang et al., 2009; Casas, 2017). If unresolved, the PERK and IRE1*α* branches of the UPR can cause inflammation and cell death (Brown and Naidoo, 2012). Therefore, we investigated the effect of B1R activation on the three ER stress sensors, ATF6, IRE1*α* and PERK in hypothalamic primary neuronal cultures. The immunoreactivity of ATF6 by immunofluorescence revealed that ATF6 was primarily observed in cytoplasm of control neurons whereas it was located within the nucleus of primary neurons treated with LDABK (300 nM) for 24 h (Figure 2A). In addition, the quantification data from western blot analysis of the lysates from primary cultures showed an increase in cleaved ATF6 protein expression by LDABK treatment at 24 and 48 h (Figure 2B). These data are further evidence that B1R stimulation induced activation and translocation of ATF6 into the nucleus. In addition, neurons treated with LDABK, showed an increase in phosphorylated IRE1*α* protein expression (Figure 2C) and an increase in phosphorylation of PERK (Figure 2D), in neurons treated with B1R agonist for 24 and 48 h. Collectively these data infer that B1R activation in neurons increased expression of ER stress sensors.

### B1R Activation Increases Phosphorylation of eIF2*α* and Splicing of XBP1 mRNA

**FIGURE 3:**
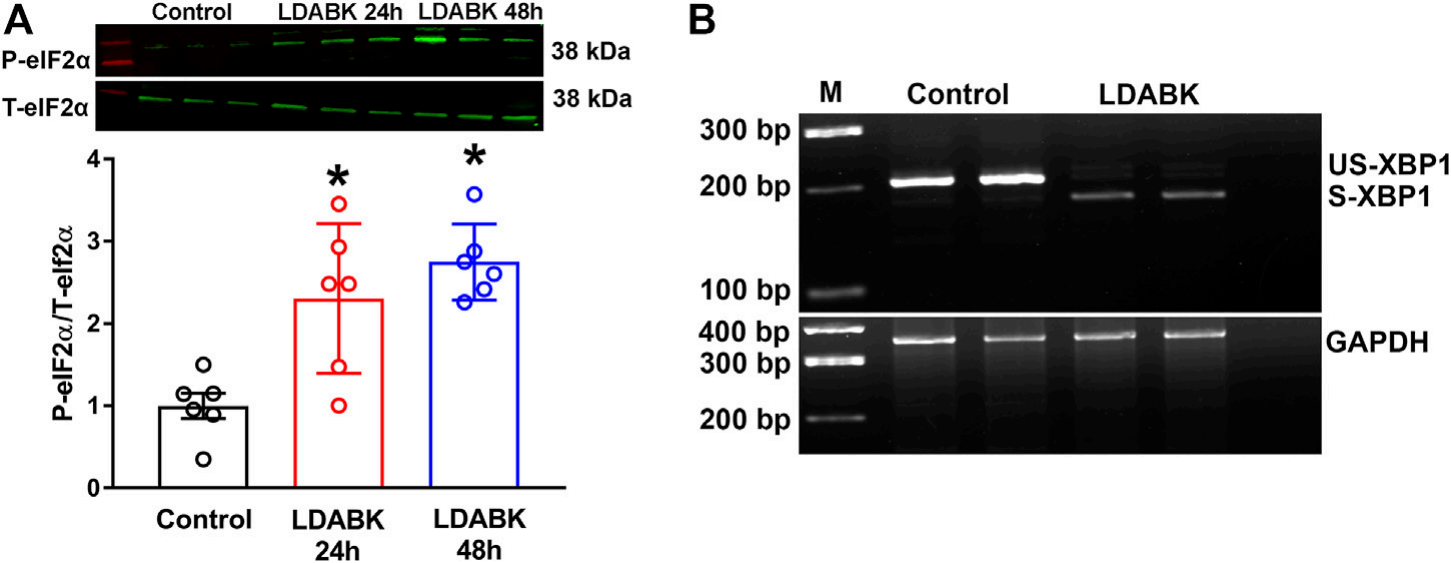
B1R activation increases phosphorylation of eIF2α and splicing of XBP1 mRNA. **(A)** Quantitative western blot analysis illustrates LDABK (300 nM) treatment increases the phosphorylation of eIF2*α* in neurons. * *p* < 0.05, compared to control. *n* = 6/group. **(B)** Agarose gel electrophoresis reveals that B1R activation by LDABK (300 nM) treatment for 24 h increases the splicing of X-box binding protein 1 (XBP1) mRNA in primary neurons. US-XBP1: Unspliced XBP1, S-XBP1: Spliced XBP1.

Since we found that B1R activation resulted in PERK phosphorylation, we next examined the phosphorylation of eIF2α, the substrate of PERK. We found that B1R stimulation significantly increased the phosphorylation of eIF2α in neurons (Figure 3A). In addition, the phosphorylated IRE1α can function as an endoribonuclease that can remove a small intron from XBP1 mRNA and sliced XBP1 can increase the expression of ER stress chaperones (Hirota et al., 2006; Junjappa et al., 2018; Adams et al., 2019). Therefore, by employing RT-PCR analysis, we determined that unspliced (inactive) XBP1 mRNA was reduced, and spliced mRNA was increased in neurons treated with LDABK, indicative of IRE1α endoribonuclease activity (Figure 3B).

### B1R Activation Increases Ca^2+^ and ATP Depletion, but Does Not Induce Cell Death in Primary Hypothalamic Neurons

**FIGURE 4:**
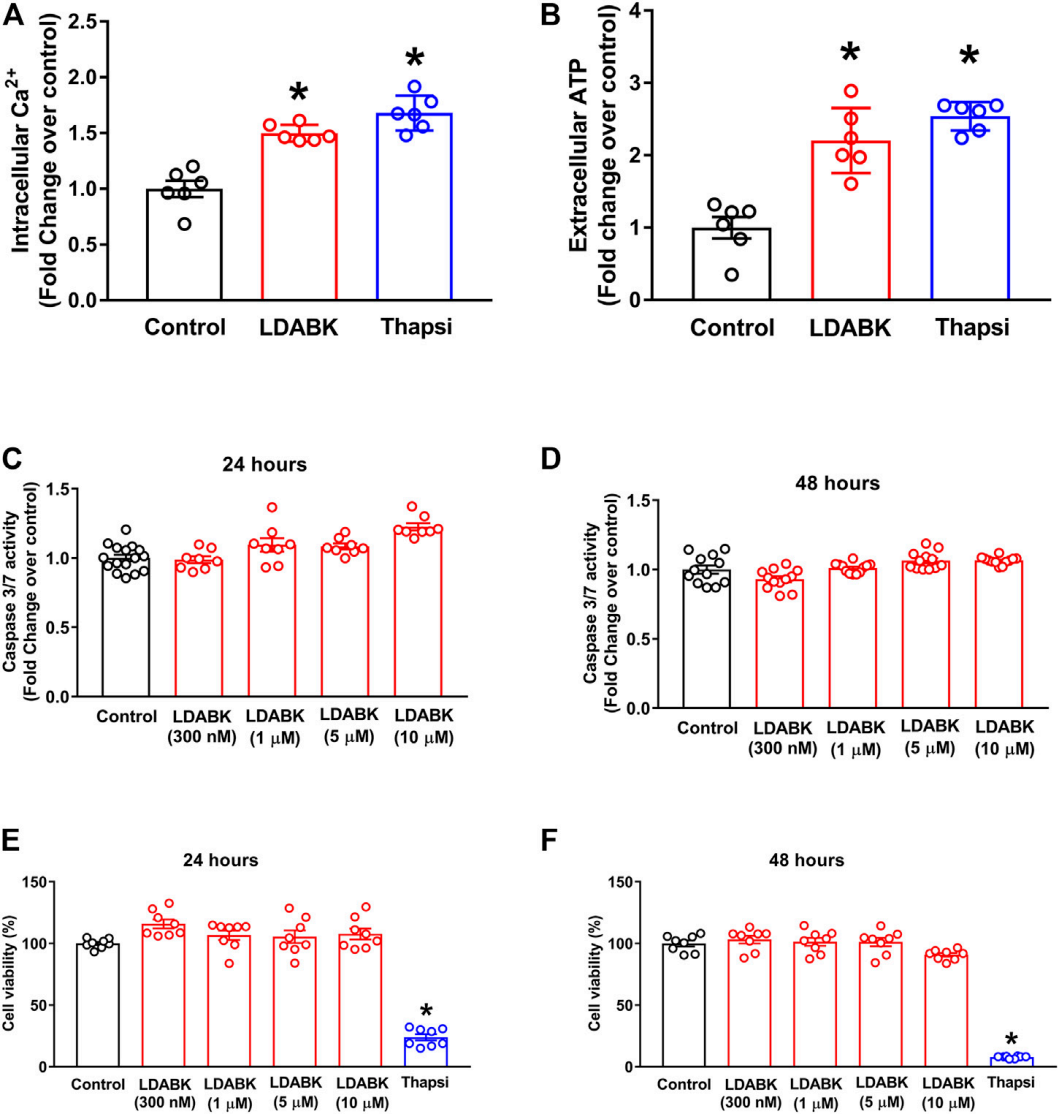
B1R activation increases Ca^2+^ and ATP depletion but does not induce cell death in primary hypothalamic neurons. LDABK (300 nM) stimulation induces an increase in intracellular Ca^2+^ after 1 h **(A)** and extracellular ATP levels after 24 h **(B)**, indicating depletion of Ca^2+^ and ATP in neurons upon activation of B1R. Thapsigargin, a known ER stress inducer, used as a positive control which increased intracellular Ca^2+^ and extracellular ATP release. LDABK treatment of neurons at doses up to 10 µM does not induce caspase 3/7 activity at either 24 **(C)** or **(D)** 48 h, indicating no increase in apoptosis. Cell viability measurements using MTS assay shows that LDABK stimulation does not induce cell death at 24 h **(E)** and 48 **(F)** hours, but thapsigargin was able to induce cell death. Thapsi = Thapsigargin. *p < 0.05, compared to control. *n* = 6–9/groups.

B1R activation results in the alteration of ER homeostasis and ultimately in severe Ca^2+^ and ATP depletion, which are essential molecules used to sustain cellular functions (Mekahli et al., 2011; Bahar et al., 2016; Su et al., 2016; Yong et al., 2019). The depletion of these energy supplies activates the UPR and causes ER stress, which, depending on the duration and severity either reestablishes ER function or leads to cell death. To determine if B1R activation results in Ca^2+^ and ATP depletion, we treated primary hypothalamic neurons with LDABK (300 nM) and measured intracellular Ca^2+^ and extracellular ATP levels. We found that activation of B1R by LDABK can significantly increase the intracellular Ca^2+^ levels in 1 h (Figure 4A) and extracellular ATP levels in 24 h (Figure 4B), indicating B1R activation can lead to Ca^2+^ and ATP depletion resulting in ER stress. We have chosen 1 h time point to measure intracellular Ca^2+^ levels to examine whether the increase in intracellular Ca^2+^ at an early time point could explain how B1R activation stimulates ER stress. Treatment with ER stress inducer, thapsigargin, also increased intracellular Ca^2+^ levels and depleted ATP levels and serving as a positive control.

In the presence of overwhelming ER stress, activated ER stress sensors can lead to oxidative stress, autophagy, or apoptosis (Qi and Chen, 2019; Elhassanny et al., 2020). To determine if B1R activation also activated ER stress-associated apoptosis, the primary hypothalamic neurons were treated with LDABK and the activity of caspase 3/7 was measured. Our data show that although B1R stimulation increased ER stress markers, caspase 3/7 activity was not increased after 24- or 48 h treatments with LDABK, suggesting that B1R activation did not induce apoptosis (Figures 4C,D). Furthermore, cell viability assay confirmed that B1R stimulation did not induce cell death in neurons treated with LDABK for 24- and 48 hours (Figures 4E,F).

## Discussion

Emerging evidence supports the concept that ER stress within the brain is a key player in blood pressure regulation (Young, 2017). The progression of hypertension can lead to the development of life-threatening complications such as heart failure, stroke, and chronic kidney disease. More recently, researchers have linked hypertension to the development of several neurodegenerative diseases (Ashby et al., 2016; Steventon et al., 2020), that may be due to the increase in sympathetic drive, oxidative stress and neuroinflammation in the cardiovascular regulatory regions of the brain that are associated with the pathogenesis of hypertension (Sriramula and Lazartigues, 2017; Mann, 2018). Recent studies have shown that defects in intracellular protein quality and age-related impairments in protein homeostasis within the brain are contributing factors in neuroinflammation and cardiovascular diseases. On the other hand, neuroinflammation has been associated with hypertension (Xia et al., 2013; Sriramula and Lazartigues, 2017), and causes organelle dysfunction, that promotes ER stress because of misfolded, unfolded or mutated protein accumulations in the ER of neurons (Yang et al., 2020). ER dysfunction can lead to apoptosis and has been implicated in the pathogenesis of cardiovascular and neurodegenerative conditions. Thus, understanding the signaling events that upregulate ER stress in the brain might provide new insights into the potential of targeting ER stress for the treatment of diseases associated with neuroinflammation including hypertension.

Previous studies from our laboratory have demonstrated that the activation of the B1R can cause neurogenic hypertension, by mediating oxidative stress and neuroinflammation in the brain (Sriramula and Lazartigues, 2017). We have validated and used primary hypothalamic neuronal cultures to study the B1R activation mediated signaling pathways in inducing inflammatory mediators and oxidative stress (Parekh et al., 2020; Parekh and Sriramula, 2020; Parekh et al., 2021). However, whether the activation of the B1R can induce ER stress is not known. We hypothesized that the B1R stimulation can induce the UPR, chronically activating the UPR signaling cascades, ultimately resulting in ER stress. To test our hypothesis, we stimulated primary hypothalamic neuronal cultures with B1R specific agonist LDABK and measured the expression of various ER stress markers and molecular chaperones. Our data suggest that, for the first time, B1R stimulation induces the activation of the UPR signaling pathways leading to the development of ER stress.

The role of B1R activation in ER stress, particularly the influence of receptor activation on mediators of the UPR is unknown. In our study, the expression of molecular chaperone GRP78, a marker of ER stress, was significantly increased in neurons by B1R activation suggesting that UPR activated, and ER stress occurred, and the mechanisms of stress protection were initiated. In response to ER stress, sensors including ATF6, IRE1α, and PERK are activated. In our study, we found that B1R stimulation of primary neurons increased translocation of ATF6 from the cytoplasm to nucleus, and increased phosphorylation of IRE1α and PERK. IRE1α activation results in autophosphorylation which then catalyzes the splicing X-box binding protein 1 (XBP1) mRNA, which results in the formation of XBP1s (XBP1 spliced), which is critical in upregulating genes involved in the UPR and inflammation. On the other hand, activated PERK undergoes autophosphorylation and then phosphorylates eIF2α, which can cause global blockade in protein synthesis to allow ER homeostasis to be reestablished (Sano and Reed, 2013). We found that B1R activation can induce phosphorylation of eIF2α and increase the splicing of XBP1 mRNA in primary hypothalamic neurons. These data further confirm that B1R activation can cause ER stress by activating all three ER-localized protein sensors. A previous study showed an upregulation of GRP78 and phosphorylated eIF2α in the rostral ventrolateral medulla, a sympathoregulatory brain region, in spontaneously hypertensive rats, occurs prior to the development of hypertension (Chao et al., 2013). This data in conjunction with the studies showing B1R upregulation in hypertension models suggest that B1R activation can induce ER stress leading to the development of hypertension.

Given its vital roles, the ER contains high concentrations of ATP and Ca^2+^, which are primarily used for protein folding and post-translational modifications (Lin et al., 2021). To perform its many functions, the ER relies on ATP to fulfill its energetic requirement for normal functioning. Physiological stressors such as a depletion of intracellular Ca^2+^ and ATP supply, can impair ER function and consequentially result in the activation of the UPR (Ma and Hendershot, 2004; Lin et al., 2008). Whether B1R activation has any role in initiating these stressors is not known. We found that primary neurons treated with B1R agonist showed an increase in Ca^2+^ and ATP levels in the media suggesting that a depletion of intracellular Ca^2+^ and ATP supply which can trigger ER stress. Unresolved ER stress within the cell can result in the activation of apoptotic pathways mediated by various proteins such as CHOP and caspases, which ultimately lead to cell death. Our data confirmed that B1R activation did not induce apoptosis or cell death in neurons despite increasing ER stressors.

Emerging evidence has indicated that ER stress is a mediator of sympathoexcitation and hypertension (Purkayastha et al., 2011; Young et al., 2015; Young, 2017). Administration of the ER stress inducer, thapsigargin, directly into the brain lateral cerebral ventricles of mice increased blood pressure response and renal sympathetic nerve activity (Purkayastha et al., 2011). Several animal models have been used previously to study the association between ER stress and hypertension including Ang II infusion, high salt-diet feeding, DOCA-salt hypertension, and SHR (Young, 2017). However, the mechanisms by which ER stress is upregulated in hypertension are still being investigated. We have previously shown that B1R expression is upregulated in the neurons of hypertensive mice, and that the chronic B1R blockade in the brain with specific B1R antagonist R715 attenuated salt sensitive hypertension along with attenuation of neuroinflammation and autonomic dysfunction (Sriramula and Lazartigues, 2017). Our recent findings further support the evidence that blocking B1R activation prevents inflammation and oxidative stress in neurons (Parekh et al., 2020). However, whether ER stress is involved in B1R mediated beneficial effects are not known. Therefore, in this study, using primary hypothalamic neuronal cultures, we tested the hypothesis that B1R activation can induce ER stress. Overall, key findings of our study are 1) B1R stimulation induces GRP78 expression in neurons; 2) B1R stimulation results in activation of the three primary ER stress sensors; 3) B1R stimulation results in increased intracellular Ca^2+^ release and ATP depletion; but 4) B1R activation does not result in apoptosis as indicated by caspase 3/7 activity and cell viability assay in primary hypothalamic neurons. Since we have shown previously that B1R activation can induce oxidative stress and inflammation within the brain hypothalamic neurons during hypertension (Sriramula and Lazartigues, 2017), ER stress induced by B1R activation might be involved in enhanced generation of reactive oxygen species and inflammatory cytokine production. B1R activation upregulated three primary ER stress sensors in neurons. However, whether B1R can activate these ER stress sensors in the brain in hypertensive animal models needs to be investigated. Further studies are needed to understand the precise role of B1R activation mediated ER stress and its contribution to the development of hypertension.

## Data Availability

The original contributions presented in the study are included in the article/Supplementary Material, further inquiries can be directed to the corresponding author.
